# *Saccharomyces cerevisiae* as a Model for Studying Human Neurodegenerative Disorders: Viral Capsid Protein Expression

**DOI:** 10.3390/ijms242417213

**Published:** 2023-12-07

**Authors:** Svetlana V. Bayandina, Dmitry V. Mukha

**Affiliations:** Vavilov Institute of General Genetics Russian Academy of Sciences, 119991 Moscow, Russia

**Keywords:** neurodegenerative diseases, *Saccharomyces cerevisiae*, protein aggregation, virus factories, IPOD, JUNQ, INQ, CytoQ, capsid virus proteins, transcriptional regulatory pathways

## Abstract

In this article, we briefly describe human neurodegenerative diseases (NDs) and the experimental models used to study them. The main focus is the yeast *Saccharomyces cerevisiae* as an experimental model used to study neurodegenerative processes. We review recent experimental data on the aggregation of human neurodegenerative disease-related proteins in yeast cells. In addition, we describe the results of studies that were designed to investigate the molecular mechanisms that underlie the aggregation of reporter proteins. The advantages and disadvantages of the experimental approaches that are currently used to study the formation of protein aggregates are described. Special attention is given to the similarity between aggregates that form as a result of protein misfolding and viral factories—special structural formations in which viral particles are formed inside virus-infected cells. A separate part of the review is devoted to our previously published study on the formation of aggregates upon expression of the insect densovirus capsid protein in yeast cells. Based on the reviewed results of studies on NDs and related protein aggregation, as well as viral protein aggregation, a new experimental model system for the study of human NDs is proposed. The core of the proposed system is a comparative transcriptomic analysis of changes in signaling pathways during the expression of viral capsid proteins in yeast cells.

## 1. Introduction

A characteristic feature of neurodegenerative diseases (NDs) is the formation of protein aggregates (inclusion bodies) in cells of the nervous system, formed as a result of the misfolding of proteins [1,2,3,4,5,6]. To reduce protein aggregation, cells have developed a complex quality control system including cellular chaperones and ubiquitin-mediated degradation by proteasomes [7,8,9]. When the capacity of this system is exceeded, proteins aggregate in intracellular inclusions. The molecular mechanisms underlying both the formation of protein aggregates and the functioning of the protein quality control (PQC) system, despite the large amount of experimental data accumulated, remain incompletely known.

Inclusion bodies formed in various NDs may consist of misfolded proteins that are completely unrelated; at the same time, the formed inclusion bodies share many common features. Importantly, similar inclusion bodies are formed in cells infected with viruses, where they appear as aggregates of electron-dense amorphous material containing viruses and intermediate assembly products called viroplasm or viral factories [10,11,12,13,14,15,16,17,18].

This review analyzes some of the results obtained in studying the formation of protein aggregates in the yeast *Saccharomyces cerevisiae*. We summarize studies of the aggregation of both proteins relevant to the development of human neurogenerative diseases and model proteins with disruptions in the amino acid sequence leading to changes in protein folding; we also consider the formation of protein aggregates under conditions of heat stress. In addition, we summarize our previously published data on the formation of protein aggregates during the expression of the densovirus capsid protein in yeast cells.

Based on the summarized experimental data, we propose a new model system for studying the molecular mechanisms of protein aggregate formation, the core of which is a comparative transcriptomic analysis of changes in signaling pathways during the expression of viral capsid proteins in yeast cells.

Note that the main objective we pursued in writing this review was to draw the attention of researchers to our proposed new model system for the study of human NDs. We believe that generalization of the results of transcriptomic analysis obtained in the study of capsid protein aggregation of different types of viruses in yeast cells will improve the understanding of both the PQC system and the molecular mechanisms of protein aggregate formation in cells of the nervous system in NDs, including the signaling pathways that determine these processes.

## 2. Brief Description of Neurodegenerative Diseases and the Experimental Models Used to Study Them

NDs are a heterogeneous group of neurological disorders that include Alzheimer’s disease, Parkinson’s disease, tauopathies, frontotemporal dementia, amyotrophic lateral sclerosis (ALS), synucleinopathies (i.e., Lewy body dementia and multisystem atrophy), Huntington’s disease (HD) and related polyglutamine diseases (including spinocerebellar ataxias), prion disease, traumatic brain injury, chronic traumatic encephalopathy, stroke, spinal cord injury, and multiple sclerosis, which affect the lives and health of millions of people (especially the elderly) worldwide. The epidemiology, symptoms, genetics, and pathological signatures of these NDs have been extensively studied and discussed in detail [5,6,19]. NDs have many common features, such as their chronic and progressive nature, increasing prevalence with age, collapse of the structure and function of neural networks, and neuron loss in the central nervous system [5,6]. Another hallmark event of most NDs is caused by the misfolding of key proteins, wherein the proteins enter a so-called amyloid state in which they are assembled into noncovalent cross-β fibrous polymers, which consequently cause cellular dysfunction [5,6].

To date, the brain regions where pathological processes occur in each type of neurodegenerative disease have been identified, the genes associated with these diseases have been described, and for most NDs, the proteins whose aggregation occurs in the cells of the nervous system of the diseased organism have been identified [19] (see Table 1).

Despite the considerable progress that has been made in the study of human NDs, the details of the molecular genetic processes underlying these diseases remain poorly understood. In particular, this is because experimental research in humans is difficult for ethical reasons. Monkeys, cats, dogs and other large animals [20], mice [21], rats [22], fishes [23], invertebrate animals (flies and worms) [24,25,26], and microorganisms—bacteria [27] and yeast [28,29,30,31]—are used as experimental models to study the pathophysiology of NDs and their treatment [32]. The issues with these models are they do not sufficiently recapitulate the human disease state. The pathological symptoms exhibited by these models are consistent with those observed in humans, but the sequence of events leading to the occurrence of pathophysiologic changes differ. New experimental models will provide an understanding of these diseases at the cellular and molecular levels, which is of fundamental importance.

In fact, many insights for understanding the molecular basis of complex human NDs caused by aberrant protein folding have been gained from model organisms, particularly yeast. The unicellular fungus known as budding yeast, *Saccharomyces cerevisiae*, has historically served as a valuable tool for uncovering many fundamental eukaryotic genetic and cellular processes. This yeast has numerous unique advantages compared to other model systems. First, it has a simple eukaryotic cellular organization and numerous conserved biological processes, such as those involved in the cell cycle, organelle function, energy metabolism, protein turnover and folding, the secretory pathway, mitochondrial biology, signal transduction, and gene interaction and recombination [33,34,35,36]. A few Nobel prizes have been awarded for discoveries made in yeast [37]. Second, it is a nonhazardous organism (GRAS status) with a short generation time (1.5 h on rich medium), so it can be easily cultured in a relatively inexpensive manner compared to other eukaryotic organisms. This allows for faster completion of experiments on a larger scale than in other systems. Third, the yeast genome was the first eukaryotic genome to be fully sequenced and assembled in 1996 [38,39]. Its genome is approximately 12.2 Mbp, with 6275 genes compactly packaged on 16 chromosomes. Sixty percent of yeast genes show significant homology with human genes, and approximately 31% of yeast genes are human orthologs. Moreover, of the 386 essential yeast genes that can be functionally replaced with human orthologs, 157 genes have disease associations in the OMIM (Online Mendelian Inheritance in Man) database [34]. This means that yeast and humans share cellular structures and molecular processes, making yeast a powerful tool to study disease-related processes.

Despite the described advantages of yeast as a model system for studying human pathologies, it should be noted that yeast can only be used as a model for studying fundamental biological processes whose molecular mechanisms are characteristic of cells of all eukaryotic organisms and are evolutionarily conserved, e.g., the PQC system and/or protein aggregation. The limitations of yeast as a model organism for studying human tissue-specific pathways are described in numerous excellent recent reviews [40,41].

## 3. Aggregation of Proteins Related to Human Neurodegenerative Diseases in Yeast Cells

This chapter will summarize the available experimental data on the aggregation in yeast cells of proteins whose aggregation has been identified in studies of human NDs, such as polyglutamine proteins, α-synuclein, TDP-4, FUS, SOD1, and tau (see Table 1). However, we will not discuss data on the Aβ protein associated with Alzheimer’s disease, as several excellent reviews have recently addressed specific aspects of Alzheimer’s diseases using yeast models [28,42,43,44,45,46]. In addition, this review will not discuss studies related to prion proteins because, in our view, prions are a special type of protein, and summarizing the results related to their study requires separate consideration.

### 3.1. Polyglutamine Proteins

There are several known human NDs caused by the expansion of CAG trinucleotide repeats in the coding region of the corresponding genes. These diseases are often called “polyglutamine (polyQ) diseases” because the CAG trinucleotide encodes the amino acid glutamine (Q). To date, glutamine expansions have been described in the following human proteins: huntingtin (Htt); Sca1, Sca2, Sca3, Sca6, Sca7, and Sca17 (different types of spinocerebellar ataxia); androgen receptor protein (spinobulbar muscular atrophy); and atrophin-1 (dentatorubro-pallidoluysian atrophy). The features of the clinical manifestations characteristic of each of these diseases, determined by the type of neurons in which inclusions formed as a result of amyloidization of the described proteins, are observed [47,48,49]. Using yeast as a model system, the aggregation of the huntingtin protein has been studied in the most detail [50].

The human huntingtin locus is large, spanning 180 kb and consisting of 67 exons [51]. This locus encodes a fairly large protein (350 kDa) that performs important, but not fully understood, functions, in particular, those necessary for the normal functioning of synapses [52]. The first exon may contain a variable number of CAG repeats, normally ranging from 6 to 35. Proteins with 36 or more repeats (up to 180 repeats have been described) tend to form amyloid aggregates, which correlates with the development of HD. Importantly, the hallmark of HD is the proteolytic production of an N-terminal fragment of the huntingtin protein (containing a polyQ repeat), which forms aggregates in the nucleus and cytoplasm of affected neurons [53,54].

To study the influence of various factors on the aggregation of the human huntingtin protein in yeast cells, single-copy plasmids containing DNA fragments corresponding to the N-terminal region (amino acids 1-68 of the wild-type protein) of the investigated protein fused to the GFP gene were generated. These vector constructs differed from each other in the number of CAG trinucleotide repeats encoding glutamine (25, 47, 72, or 103 residues). Expression was performed under the control of a strong constitutive yeast promoter (GPD). The results showed that the degree of aggregation varied with polyQ repeat length; in particular, a protein containing 25 glutamine repeats showed no signs of aggregation, whereas when a protein containing 103 glutamine repeats was expressed, one large aggregate was formed [55]. Mutations in yeast cells inhibiting the ubiquitin/proteasome pathway did not affect the aggregation of the studied protein fragments in the series of experiments. At the same time, changes in the expression level of chaperone proteins, in particular, overexpression of Hsp40, Hsp70, and Hsp104, modulated the aggregation of fragments containing 72 and 103 glutamine repeats [56,57].

In the amino acid sequence of the huntingtin protein, a proline-rich region is located next to the polyQ site. Deletion of this proline-rich region effects on the number and shape of formed aggregates [58,59].

Interestingly, when the dependence between the intensity of huntingtin protein aggregate formation in yeast cells and the age of the cell culture was studied, the results showed that proteins containing 25Q did not aggregate during the logarithmic stage of cell growth or in aged cells. Proteins containing 103Q aggregated in both young and aged cells, while proteins containing a moderate amount of glutamine (47Q) aggregated only during cell culture senescence. Mutations in two genes (SIR2 and HSF1), the expression levels of which correlate with aging, affected the dynamics of huntingtin protein aggregation in yeast cells [60].

Currently, when yeast is used as a model organism, transcriptomic, proteomic, and metabolomic analyses are the most promising modern methodological approaches for understanding the molecular mechanisms of the formation of aggregates of proteins containing polyQ tracks, particularly the huntingtin protein [61,62,63,64,65].

### 3.2. α-Synuclein

Parkinson’s disease, certain forms of dementia, and multisystem atrophy are characterized by the accumulation of aggregated α-synuclein in protein inclusions called Lewy bodies (LBs) [66]. This class of ND is generally referred to as synucleinopathies [67,68]. It is currently unclear whether the formation of LBs causes cell pathologies or acts as a protective cellular mechanism, leading to the inactivation of soluble prefibrillar oligomeric forms of α-synuclein [69,70,71,72,73].

Under physiological conditions in human cells, α-synuclein is considered a presynaptic protein [74] associated with vesicles and membranes [75]. However, its exact function is not yet well understood [76]. Several studies have suggested that α-synuclein plays a critical role in enabling vesicle fusion; others have reported a chaperone-like activity [77,78,79,80].

Human α-synuclein is encoded by the SNCA gene. The protein consists of 140 amino acids and has a molecular mass of 17 kDa. The protein has N-terminal imperfect repeats based on the amino acid motif KTKEGV. The central region of α-synuclein is strongly hydrophobic, leading to protein dimerization [81,82]. The N-terminus of α-synuclein is critical for oligomerization [83], whereas the C-terminus is highly acidic, facilitating protein–protein interactions [84] and possibly inhibiting aggregation [85]. The α-synuclein protein is intrinsically unfolded; in solution, it lacks a stable tertiary structure and instead forms α-helices, β-sheets, and various complex multimeric structures, such as fibrils, fibers, and aggregates [86].

Multiple wild-type alleles at the SNCA locus, such as duplications or triplications, have been shown to be associated with the familial form of the disease [87,88]. In addition, three missense mutations, A30P, A53T, and E46K, correlate with the autosomal dominant early form of Parkinson’s disease [89,90]. These mutations render α-synuclein more prone to forming amyloid fibrils [91].

It has been shown that α-synuclein in human nerve cells can undergo various posttranslational modifications, namely, phosphorylation [92], ubiquitination [93,94], SUMOylation [95], acetylation [96], nitration [97], and/or glycosylation [98]. Posttranslational modifications of α-synuclein are known to affect its toxicity and ability to form aggregates, but the exact contribution of different posttranslational modifications to the disease mechanism is still unclear.

There is no homolog of the human SNCA gene encoding α-synuclein in the yeast genome. It has been shown that expression of the human α-synuclein gene in yeast affects vesicle trafficking [99,100,101], induces oxidative stress [102], and causes mitochondrial dysfunction [103,104].

It has been demonstrated that when the human α-synuclein gene is heterologously expressed in yeast cells, the resulting protein is transported via the secretory pathway to the plasma membrane, where it forms small aggregates [105,106]. As the level of α-synuclein expression increases, its localization changes significantly, leading to the formation of cytoplasmic inclusions similar to the LBs found in Parkinson’s disease neurons. This is accompanied by an increase in toxicity, defined as reduced cell growth followed by cell death. The increase in toxicity caused by α-synuclein is dependent on dosage. Studies have shown that the expression of three copies of the wild-type α-synuclein gene or two copies of the α-synuclein gene with the A53T mutation under the control of the yeast GAL1 promoter leads to growth inhibition and protein aggregation [107].

### 3.3. TDP-4, FUS, and SOD1

Like most NDs, ALS and frontotemporal lobar degeneration (FTLD) are characterized by the misfolding and aggregation of proteins in affected neurons [108]. The aggregates formed in these diseases contain proteins such as TDP-43 (trans-activation response DNA-binding protein 43), FUS (fused in sarcoma), SOD1 (superoxide dismutase), and C9orf72 that are well-known pathologic hallmarks of ALS/FTLD [109,110,111,112,113,114,115,116]. Using yeast as a model system, the aggregation of TDP-43, FUS, and SOD1 has been studied in the most detail. It was shown that the expression of these proteins in yeast reproduces the main features of ALS/FTLD pathology, including aggregation, protein mislocalization, and cellular toxicity [117,118,119,120].

FUS and TDP-43 have similar domain structures and serve many similar functions. TDP-43, which is 414 amino acid residues in length, has been shown to bind to both DNA and RNA and has multiple functions in transcription repression, pre-mRNA splicing, and translation regulation. FUS, which is 526 amino acid residues in length, plays an important role in various cellular processes, such as the regulation of transcription, RNA splicing, RNA transport, and DNA repair. In neuronal cells, it plays a critical role in dendritic outgrowth formation and stability, RNA transport, mRNA stability, and synaptic homeostasis [121].

Both of these proteins have highly conserved RNA recognition motifs (RRMs) and glycine-rich domains [121]. Moreover, using a bioinformatics algorithm originally developed to identify yeast prion domains [122], “prion-like” domains were identified in the N-terminal part of FUS (amino acids 1–239) and in the C-terminal part of TDP-43 (amino acids 277–414) [121,122]. Similar to the prion domains of yeast prion proteins such as Sup35, Ure2, and Rnq1, these domains are rich in uncharged polar amino acids [123,124].

Expression of human FUS and TDP-43 proteins in yeast leads to cytoplasmic aggregation of these proteins and toxicity, thus modeling key aspects of ALS- and FTLD-related proteopathies. The RRM and prion-like domains have been shown to be key to the aggregation of the proteins studied. The patterns of localization of FUS and TDP-43 protein aggregates in yeast cells in terms of size, shape, and number of foci in the cytoplasm are strikingly similar. Indeed, when FUS-YFP and TDP-43-CFP were coexpressed in the same cell, the fluorescence signals colocalized in the same cytoplasmic foci [121].

The aggregates formed by FUS and TDP-43 in yeast cells have been shown not to react with amyloid diagnostic dyes and are SDS-soluble. Thus, FUS and TDP-43 in yeast cells form aggregates that are probably not amyloid in nature, as are aggregates of these proteins observed in patients with ALS and FTLD [121,125,126].

To date, many mutations in the human FUS and TDP-43 genes have been described and have been associated with some familial and sporadic ALS cases [127]. Using a yeast model, the influence of these mutations on the aggregation and toxicity of the proteins under study was tested. The mutations in TDP-43 significantly increased the degree of aggregation and toxicity of this protein in yeast cells. Moreover, almost all ALS-linked mutations in the TDP-43 gene are located in the DNA sequence encoding the prion-like domain of this protein. However, ALS-linked FUS mutations do not promote FUS aggregation in yeast [121].

Superoxide dismutase 1 (Sod1) is an enzyme that, in humans, is encoded by the SOD1 gene located on chromosome 21. Sod1 is 154 amino acid residues long, and alternative splicing results in five forms of the enzyme that differ in their localization in the human body [128]. This enzyme is a 32 kDa homodimer containing an intramolecular disulfide bond and a binuclear Cu/Zn site in each subunit. SOD1 binds copper and zinc ions and is one of three superoxide dismutases responsible for the destruction of free superoxide radicals in human cells. SOD1 is a soluble cytoplasmic and mitochondrial intermembrane protein that converts naturally occurring but harmful superoxide radicals into molecular oxygen and hydrogen peroxide. Recently, it has been shown that under oxidative stress, SOD1 can localize not only in the cytoplasm but also in the nucleus [129,130]. Mutations in this gene (more than 150 identified to date) are associated with familial ALS1 [131]. The protein (both wild-type and ALS1 variants) has a propensity to form fibrillar aggregates in the absence of intramolecular disulfide bonding or bound zinc ions. These aggregates can exert cytotoxic effects. Zinc binding promotes dimerization and stabilizes the wild-type form.

Yeast has been shown to have a homolog of the human SOD1 gene. Expression of the human SOD1 gene in yeast cells lacking the homologous gene showed that human SOD1 can fully complement the biological function of yeast SOD1 [132]. A series of studies have been conducted in which the wild-type human SOD1 gene and SOD1 genes containing various ALS-related mutations were expressed in yeast cells [133,134,135]. Interestingly, neither the wild-type human protein nor any of the mutant variants of this protein corresponding to ALS-related mutations in yeast cells caused impairment of the cell culture growth rate [133,134,135,136,137]. However, it has been shown that the wild-type SOD1 protein, as well as ALS-associated SOD1 mutants, tends to selectively aggregate near mitochondria, where the protein may exert toxic effects that are not yet fully understood [120]. It has been shown that the aggregates formed are SDS-soluble and of a nonamyloid nature [132,138].

Integrated transcriptomic and metabolomic analysis is the most recently developed and promising methodological approach that uses yeast as a model system to understand the biological role of the described proteins in the deregulation of pathways associated with the disease. To date, only preliminary, but encouraging, results have been obtained, and in our view, further research is warranted [139,140].

### 3.4. Tau

Tau, also known as microtubule-associated protein tau (MAPT), is expressed predominantly in cells of the nervous system. The main function of this protein is the stabilization of microtubules, which occurs through a kiss-and-hop mechanism [141,142]. The human MAPT gene contains 16 exons. In cells of the nervous system, alternative splicing yields six tau isoforms, ranging in size from 352 to 441 amino acids. The molecular mass of the tau isoforms ranges from 48 kDa to 68 kDa [143]. Numerous human NDs have been shown to be associated with tau protein aggregation (see Table 1). Although tau is a “naturally unfolded protein”, conformational changes have been detected and could be essential in the formation of aggregates [144]. Various posttranslational modifications of the tau protein (phosphorylation, acetylation, ubiquitination, SUMOylation, methylation, glycosylation, glycation, proteolysis, and numerous others) occur in the brain cells of both healthy people and patients with NDs [145]. Posttranslational modifications play an important role in tau protein aggregation, and phosphorylation is of particular importance.

Expression of tau in yeast does not result in a negative phenotype [146]. Tau protein expressed in yeast under normal conditions, either under mild proteotoxic stress or in mutants with impaired proteotoxic stress response pathways, did not result in toxicity or the formation of obvious aggregates. In chronologically aged cells, no appreciable Tau aggregates were formed [147].

In *S. cerevisiae*, tau phosphorylation and hyperphosphorylation are under the control of two protein kinases, Mds1 and Pho85, which are orthologs of two human protein kinases, GSK3β and cdk5, respectively. It has been shown that when tau is expressed in yeasts lacking Pho85, the tau phosphorylation and aggregation levels increase, indicating that Pho85 (and possibly cdk5 in human neuronal cells) is a negative regulator of tau phosphorylation. On the other hand, deletion of the Mds1 gene leads to a decrease in the amount of hyperphosphorylated tau protein and, accordingly, to a decrease in the amount of the aggregated form of this protein. Thus, the tau protein expressed in yeast lacking Pho85 undergoes posttranslational modification by phosphorylation and, as a consequence, changes its conformation and forms aggregates. The hyperphosphorylation and aggregation of the tau protein in yeast cells is similar to processes that are characteristic of neurodegenerative tauopathies, including Alzheimer’s disease [148].

### 3.5. Screening of the Compounds Preventing Aggregation and Toxicity of Disease-Specific Proteins

The yeast model systems described above, which allow the study of the aggregation of proteins whose aggregation was originally identified in studies of human NDs, can be used to screen potential drugs that prevent the aggregation and toxicity of these proteins. Indeed, yeast has been shown to be a very useful platform for drug screening to identify potential therapeutic molecules. Importantly, many of these results initially obtained in yeast have subsequently been confirmed in mammalian systems, confirming yeast as reliable. For review, see, e.g., [28,33,149,150]. To our knowledge, the most representative candidate drugs identified using *S. cerevisiae* models for amyloid-associated diseases are summarized in the review [28].

Although yeast cells are well-suited for high-throughput screening in search of chemical compounds that inhibit aggregation and/or toxicity, one limitation is the inability of many drugs to cross cell membrane and/or cell wall barriers or to accumulate within the cell. This problem can be overcome by using yeast strains containing mutations in the ERG6 or PDR5 genes. Erg6 mutants have increased membrane permeability; pdr5 mutants more easily accumulate drugs intracellularly. For review, see, e.g., [33].

## 4. Misfolded Proteins Are Sequestered in Spatially Distinct Subcellular Compartments in a Yeast Model System

**Figure 1:**
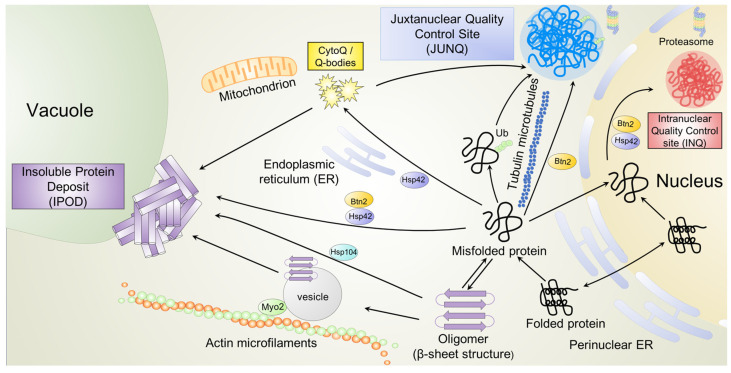
This paper demonstrates the main types of cellular inclusions and the basic factors known to play a role in their formation. Misfolded proteins are recognized by the yeast chaperone machinery—Hsp42, Hsp104, and Btn2 are shown as the main players. These chaperones are important for sequestering misfolded proteins into quality control sites: the juxtanuclear JUNQ, intranuclear INQ, CytoQ/Q-bodies, and perivacuolar IPOD sites. Actin microfilaments and vesicle trafficking, which are regulated by the Myo2 motor protein, may be needed for IPOD formation. Tubulin microtubules may be needed for JUNQ formation. The localization of proteasomes, mitochondria, endoplasmic reticulum, nucleus, and vacuole are highlighted. See text for more details.

Misfolded proteins can acquire toxic conformations, disrupting fundamental cellular processes and causing some human diseases. It is suggested that the accumulation of these toxic protein conformers can be an underlying cause of age-related NDs [151,152,153]. The *S. cerevisiae*-based cell model is a critical tool for recapitulating and monitoring central aspects of protein misfolding and the formation of damaging conformers and their associated toxicity, especially in the context of aging, due to the evolutionarily strong conservation of the PQC system between yeast and mammalian cells [154,155]. PQC includes molecular chaperones, the ubiquitin/proteasome-dependent protein system (UPS), protein deposition sites, and endolysosomal machinery that consistently monitor and maintain the conformational state of cellular proteins [8,156,157]. Under severe stress or during aging, PQC homeostasis is impaired, so cells sequester and organize aberrant oligomers into distinctly localized aggregates/compartments/deposits/inclusions [8,158,159]. In eukaryotes, various PQC deposition sites have been identified and described, including major (i) cytoplasmic sites, such as Q-bodies/CytoQ/stress foci, the perinuclear quality control site (JUNQ—JUxta Nuclear Quality control compartment), or insoluble protein deposits (IPOD) and (ii) intranuclear quality control sites (INQ) [155,158,160,161,162,163,164,165,166] (see Figure 1). 

Note that previously described inclusions such as the aggresome [12,13] and the ER-associated compartment (ERAC) [167] have features in common with the newly discovered JUNQ compartment [168]. Spatial segregation of aberrant proteins into PQC deposition sites is evolutionarily conserved and occurs in yeasts and mammalian cells [160,168,169].

A variety of verified model fluorescent misfolded substrates of different origins are used to monitor inclusion formation and localization in *S. cerevisiae* (Table 2) [155,164]. In the context of this paragraph, we will not consider prions (i.e., Rnq1, Ure2, Sup35, etc.) or proteins associated with NDs as model proteins, as described in the previous chapter. As presented in Table 2, model substrates can be divided into two groups: (i) those that aggregate under thermal stress (thermolabile reporters, 37 °C and above) and (ii) those that are constitutively unfolded in yeast cells (nonthermolabile reporters) [155,170].

During heat shock and/or proteasome impairment (using MG132 proteasome inhibitor or expression in strains with deletions of genes involved in the degradation pathway), the analyzed substrates should misfold, aggregate, and accumulate at the known quality control sites. However, these substrates have numerous disadvantages for PQC research, especially when using the modern transcriptomic analysis method to study the genetic mechanisms of sequestration in depth. The nonthermolabile reporters are cleared via the UPS within 1–2 h for a variety of reasons, so scientists need to use a combination of chemical and genetic approaches to inhibit proteolysis, which may lead to physiological imbalance [155,171]. During heat shock, the transcription of many genes undergoes quantitative changes. Additionally, a change in the carbon source to switch to an inducible promoter (such as GAL1) affects the physiology of the cell.

Given the above challenges, the task of searching for model substrates that could aggregate under unstressed conditions and affect as few cellular molecular processes unrelated to aggregation as possible remains relevant. Such protein substrates will allow “clean” transcriptomic analysis and, perhaps, will elucidate new mechanisms of spatial sequestration and provide an opportunity for the development of new approaches to treat diseases associated with the aggregation of misfolded proteins.

Misfolded cytoplasmic proteins accumulate into distinct inclusion bodies depending on their properties, their chaperone-cochaperone interactions, their aggregation state and, possibly, their ubiquitination state [172,173]. Insoluble, terminally aggregated, often amyloidogenic proteins are irreversibly sequestered to the IPOD [160,165,174,175]. In contrast, the more soluble misfolded proteins targeted to the active site JUNQ are thought to either undergo chaperone-mediated refolding or are eliminated via the UPS [158,159,160,164,176,177].

Early during misfolding, most cytosolic proteins form small, dynamic aggregates (called Q-bodies, CytoQ, or stress foci), some of which are not associated with organelles [170,176,178], while others are associated with organelles, e.g., the endoplasmic reticulum (ER) [161], mitochondria [163,179,180], and vacuoles [163]. Their formation and movement are cytoskeleton-independent but require a cortical ER and the small heat shock protein Hsp42 [161]. Hsp42 acts as a first line of defense during unfolding stress and associates first with a broad range of misfolded proteins [166,176,181]. Under prolonged proteotoxic stress, Q-bodies could coalesce, perhaps along the endomembrane system, into the JUNQ. Moreover, Q-bodies may be directly linked to the envelope protein complex II (COPII)-dependent transport system and, as a result, may end up near the vacuole, the site of IPOD deposition [163,173].

Originally, it was suggested that ubiquitination of model substrates acts as the sorting principle determining JUNQ deposition and their subsequent elimination [160,182]. The yeast prion Rnq1, which is sequestered exclusively in the IPOD, partially accumulates at the JUNQ site when fused to ubiquitin. It was also demonstrated that JUNQ formation was reduced when the deubiquitinating enzyme Ubp4 was overexpressed or two E2 ubiquitin-conjugating enzymes (Ubc4 and Ubc5) were lacking [160]. Another study showed that sorting of some misfolded substrates occurs independently of ubiquitination. Miller and colleagues [178] showed that misfolded substrates ubiquitinated in vivo by San1 and Ubr1 (two main yeast E3 ubiquitin ligases) accumulate in both JUNQ and CytoQ, even in the nonubiquitinated state (i.e., in *ubr1Δ san1Δ* double mutants). In general, these results indicate strong substrate-specific differences in the role of ubiquitination as a sorting signal.

Interestingly, it was shown that the small chaperone Btn2 (yeast homolog of the mammalian HOOK proteins) also has an established role in the partitioning of misfolded proteins among INQ, JUNQ, and IPOD. Btn2 can either directly interact with Hsp42 and direct nonamyloid substrates to IPOD in the cytoplasm or to INQ in the nucleus [159,164,166]. Btn2 can also bind to chaperones, promoting the sorting of misfolded proteins to JUNQ [178,183]. These data emphasize the specific interaction between substrates and chaperones in spatial sequestration.

PQC deposition sites are also observed in the nucleus near the nucleolus, as nuclear misfolded proteins and some transported cytoplasmic proteins are sequestered in the membraneless compartment INQ [166]. Like JUNQ, INQ is more amorphous with rapid protein turnover due to enrichment in the 26S proteasome [159,166]. Two small chaperones (Btn2 and Hsp42) that act as sequestrates recognize and bind to the exposed hydrophobic surfaces of aberrant proteins and sequester them to the INQ. Similar to other small chaperones, Btn2 has an alpha-crystallin-like domain (aCLD) flanked by an N-terminal domain (NTD) and a C-terminal domain (CTD). Btn2′s NTD is not involved in INQ formation, but rather acts as a recruitment domain for disaggregates that aid in INQ solubilization. Instead, both the aCLD and CTD of Btn2 are essential for INQ formation. In particular, the aCLD acts as the substrate-binding site [166].

In a new study, Sontag and colleagues [173] unequivocally demonstrated nuclear localization of the INQ and cytosolic localization of the JUNQ. This finding is important because it is still unknown whether JUNQ and INQ are identical or independent structures, since both were discovered and described using the same model protein substrates and under similar conditions [165,166]. The formation of both compartments is independent and coordinated for localization to opposite sides of the nuclear envelope (NE). It was also shown that the homing process is coordinated by nuclear pores and a perinuclear endosomal sorting complex required for transport (ESCRT) pathway [184,185,186]. These data represent an important alternative pathway for the controlled clearance of misfolded proteins and link PQC with NE quality control [173,187].

Insoluble protein deposition (IPOD) is a peripheral vacuolar-associated protective compartment in which β-sheet-rich amyloid proteins and prions are terminally isolated [159,160,165,172,174,175,188]. Amyloids are insoluble, highly ordered, fibrous aggregates with very high levels of β-strands oriented perpendicularly to the fibril axis. Their occurrence underlies several fatal NDs (see Table 1). However, the IPOD could also store nonamyloid substrates—as noted above, Btn2 can directly interact with Hsp42, directing the nonamyloid substrates to the IPOD [159,160,164,165,178,189]. Amyloid aggregates are targeted to their perivacuolar site via myosin 2-based vesicular transport [165,173,175]. Moreover, all known amyloid-based cytosolic yeast prions require Hsp104 for targeting the IPOD [190,191,192]. Amyloids accumulate constantly at the IPOD without any external stress when overproduced [159,165,189].

The underlying intracellular localization and ultimate fate of misfolded proteins are still insufficiently understood, but all these data indicate that sequestration of these oligomers is a nonrandom process that is directed and regulated by multiple additional factors, the identity and nature of which differ.

We would like to emphasize that it seems unusually important and interesting that Q-body formation apparently occurs independently of cytoskeletal elements [161], while JUNK formation is apparently microtubule dependent [193], and actin-myosin microfilaments can play a key role in IPOD formation [165,174,175].

In general, the accumulation of harmful oligomers in compartments protects the remaining cellular milieu from toxicity, thereby reducing the burden on other PQC pathways. Subcellular sequestration also promotes asymmetric inheritance of misfolded proteins upon division so that daughter cells are free from aggregates and have a fully functional proteome, which allows cell rejuvenation [165,169,172,189,194,195]. These events are essential for eukaryotic cell survival and longevity.

The main types of cellular inclusions and the basic factors known to play a role in their formation are summarized in Figure 1.

## 5. Aggresomes and Viral Factories

The replication of viral genetic material and the formation of mature viral particles occur inside the cells of living organisms. It has been shown that this occurs not in the entire cell but in strictly defined compartments (viral inclusions) called viral factories. For review, see, e.g., [14,15,16,196,197,198]. DNA viruses form aggresome-like structures—depending on the type of DNA virus, the formation of aggresome-like structures can occur either in the cytoplasm or inside the nucleus. For review, see, e.g., [16,196,199,200,201]. RNA viruses can utilize the cellular mechanism of autophagy, leading to the formation of double-membrane vesicles in the cytoplasm. For review, see, e.g., [202,203,204,205,206].

In the context of developing new models for studying the molecular genetic processes underlying NDs, viral factories formed by DNA viruses are of the greatest interest, in our opinion.

To our knowledge, the similarity between aggresomes formed in eukaryotic cells in response to protein misfolding and viral factories formed during virus development inside the cells was first pointed out by Thomas Wileman in his review article “Aggresomes and Pericentriolar Sites of Virus Assembly: Cellular Defense or Viral Design?” [196]. In particular, the author noted that “the similarity between aggresomes and virus inclusions raises the possibility that viruses use aggresome pathways to concentrate cellular and viral proteins to facilitate replication and assembly, but alternatively, aggresomes may be part of an innate cellular response that recognizes virus components as foreign or misfolded and targets them for storage and degradation”.

Obviously, if it is true that eukaryotic cells can recognize viral proteins as foreign or misfolded, then viral proteins, in particular, capsid proteins of different virus species, must have common structural characteristics (apparently necessary for capsid formation) that are recognized by the cell as potentially dangerous. That is, we can assume that cells of eukaryotic organisms possess some kind of evolutionarily ancient defense mechanism that originally arose for defense against viruses but then lost its direct purpose because, over the course of viral evolution, viruses adapted to this defense mechanism. In other words, over the course of evolution, viruses learned to use this protective cellular mechanism to their advantage by concentrating the necessary proteins and viral genetic material in a limited space. The described antiviral defense mechanism should be evolutionarily and functionally related to the PQC system.

As described above, during PQC implementation, chaperones recognize the misfolded protein and form the corresponding protein complex; in the next step, the misfolded proteins either return to their native structure, are degraded under the action of proteasomes, or aggregate into cellular inclusion bodies. According to our hypothesis, on which we plan to base our future research, PQC and evolutionarily ancient cellular antiviral defenses have common properties and are based on similar molecular genetic mechanisms.

The molecular mechanisms underlying the cellular antiviral defense mechanism based on the cellular recognition of certain structural elements characteristic of virus proteins remain unknown. However, it is clear that the similarity of the structure of viral proteins is hardly due to the similarity of amino acid sequences, but rather due to the similarity of the tertiary/quaternary structures of the protein molecules [207]. Note that the molecular mechanisms by which the cell distinguishes a “correctly” folded protein from an “incorrectly” folded protein also remain unclear.

In the previous chapters of this review, we have shown that yeast is a convenient experimental model for studying the molecular mechanisms of thermolabile and/or constitutively unfolded protein aggregation in yeast cells. In addition, proteins whose aggregation leads to human NDs have also been shown to form aggregates when expressed in yeast cells. We believe that analysis of the formation of aggregates of capsid proteins of viruses foreign to yeast in yeast cells will allow us to reveal previously undescribed molecular mechanisms underlying the evolutionarily ancient antiviral defense of eukaryotic cells and extrapolate the results obtained to the occurrence and course of human NDs.

In our pilot experiments, the results of which were published in the Russian-language journal Genetika (Moscow) [208], we investigated the intracellular localization of the native capsid protein VP3 of German cockroach *Blattella germanica*, densovirus (BgDV1), and its mutant form during expression in *Saccharomyces cerevisiae* yeast cells.

BgDV1 was first detected at the end of the last century in a cockroach colony (P6) that originated from a natural population in a pig farm in North Carolina, USA [209]. To date, the nucleotide sequence of this virus has been described, and the expression strategy of its genetic material has been studied [210]. In particular, it has been shown that the capsid of BgDV1 consists of three proteins, VP1, VP2, and VP3, with molecular masses of 100, 80/85, and 56 kDa, respectively. All three proteins share a similar short amino acid domain corresponding to both a nuclear localization signal (NLS) and a ubiquitination site (UbiSite) [210,211].

**Figure 2:**
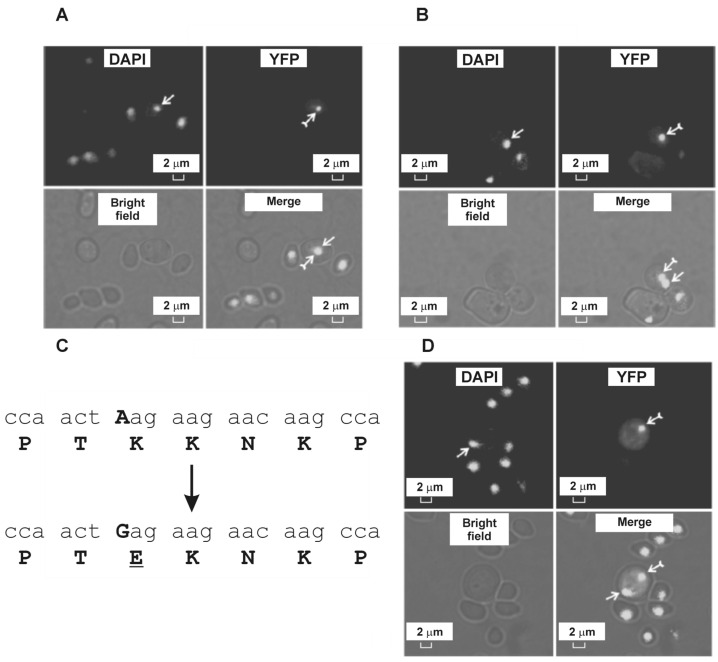
Intracellular localization of native (**A**,**B**) and mutant (**D**) forms of the YFP-VP3 fusion protein expressed in the yeast *Saccharomyces cerevisiae*. Simple arrows indicate intracellular localization of nuclei and feathered arrows indicate the localization of aggregates of the YFP-VP3 protein. (**C**) Scheme of site-directed mutagenesis leading to the elimination of the nuclear localization/ubiquitination signals of the VP3 protein. Bold capital letters indicate nucleotides that were changed, and underlined letters indicate amino acids that were changed. Source: [208].

Figure 2A,B depict the intracellular localization of the YFP-VP3 fusion protein with the native NLS/UbiSite of VP3. The newly synthesized protein is not evenly distributed throughout the cells but forms clear aggregates. A characteristic feature of the described aggregates is their colocalization with the nucleus. In most of the cells, the localization of YFP-VP3 and the cell nucleus coincided (Figure 2A); in some cells, the fluorescence signals of YFP and DAPI were in close proximity to each other (Figure 2B).

In the next stage of the study, we performed site-directed mutagenesis of the nucleotide sequence corresponding to the recombinant YFP-VP3 protein, leading to the described NLS and UbiSite not being defined as functional protein domains by bioinformatic methods. The scheme of site-directed mutagenesis is shown in Figure 2C. The intracellular localization of the mutant YFP-VP3 protein is presented in Figure 2D. The mutant form of the protein, as well as the protein with the native NLS/UbiSite, forms aggregates, but unlike the native protein, aggregates with a disturbed NLS/UbiSite are localized in the cytoplasm without any association with the nucleus.

The results obtained in the described pilot experiments, in our opinion, look very encouraging and suggest that continued research in this direction will reveal new features underlying protein aggregation. First, when YFP is expressed in yeast cells, the synthesized protein is uniformly distributed throughout the cell cytoplasm; when YFP-VP3 is expressed, aggregates are formed. Analysis of the intracellular localization of fusion proteins consisting of YFP and VP3 fragments of different lengths will, in our opinion, allow us to identify the amino acid sequence of VP3 that is necessary and sufficient for the formation of aggregates. Second, single-nucleotide substitution disrupting NLS/UbiSite leads to a change in the intracellular localization of the formed aggregates. It can be hypothesized that the aggregates formed by native YFP-VP3 molecules are JUNQ-like structures, while the mutant protein forms IPOD-like aggregates. Comparative transcriptomic analysis will, in our opinion, allow us to reveal the molecular mechanisms of bifurcation that determine the direction of movement of the studied proteins inside the yeast cell and the types of aggregates formed. In addition, there are certainly more capsid proteins of different types of viruses that should be investigated.

## 6. Conclusions and Future Perspectives

To prevent the occurrence of, or effectively treat, human NDs, it is necessary to understand the molecular genetic processes underlying protein aggregation. The use of model organisms, particularly the yeast *Saccharomyces cerevisiae*, plays an important role in the study of these processes. Yeast and humans share common cellular structures and many common molecular genetic processes underlying the functioning of cells of living organisms, which makes yeast a powerful tool for studying the processes associated with neurodegeneration.

One of the effective modern methodological approaches for studying molecular processes underlying any human disease using any model organism is transcriptomic analysis, which allows the detection of changes in gene expression that lead to the onset of pathology and its development. We note that this type of analysis is, in our opinion, the most appropriate approach to elucidate the molecular processes underlying the recognition by the cell of both its own misfolded protein and the viral capsid protein heterologous to the organism, as well as the signaling pathways that determine the type of protein aggregate formed, e.g., JUNQ or IPOD. The most important requirement for this type of analysis is the creation of an experimental model that allows the results obtained to be interpreted as clearly as possible, i.e., a model that minimizes any detectable changes in gene expression not related to the process under study.

When yeast is used as a model organism to study the processes of neurodegeneration (and protein aggregation in a broader sense), the main methodological approach at present is to analyze the expression of either specially selected reporter genes or human genes whose protein products form aggregates in the process of NDs.

The main disadvantage of the analysis of the expression of human genes whose protein products aggregate in the process of NDs, in our opinion, is the fact that these proteins can potentially exhibit in yeast cells biological activity associated not only with the process of aggregate formation but also activity characteristic of their biological function in human cells.

Specially selected reporter genes (see Table 2) used in the analysis of protein aggregation in yeast cells can be divided into two groups: the first group includes proteins that aggregate under heat stress, and the second group includes proteins that have an unstable three-dimensional structure (nonthermolabile reporters). Both types of proteins have significant disadvantages for analyzing protein aggregation by transcriptomic analysis. Heat shock induces systemic changes in the expression of the yeast genome; when nonthermolabile reporter genes are used, chemical and genetic approaches usually have to be used to suppress proteolysis, which also complicates the interpretation of transcriptome analysis results.

Based on the aforementioned assumption of the similarity between viral factories (viral inclusions) and inclusion bodies formed in eukaryotic cells in response to protein misfolding, we have proposed a new experimental model that essentially consists of analyzing the aggregation of viral capsid proteins in yeast cells. The proposed new experimental model was tested in a series of pilot experiments on the expression of insect densovirus capsid protein in yeast cells [208]. The expressed protein formed aggregates, and the localization of these aggregates relative to the cell nucleus was dependent on the presence/absence of ubiquitination of the expressed capsid protein. In other words, the aggregation process in this model was not a consequence of “random self-assembly” of virus-like particles, but rather a process under the control of yeast cells.

Note that the study of the molecular processes underlying the aggregation of virus capsid proteins in yeast cells is devoid of the abovementioned drawbacks inherent in studies using human neurodegenerative proteins or reporter proteins under heat shock conditions, as well as the study of nonthermolabile proteins using proteolysis inhibitors. It can be assumed that capsid proteins of viruses do not fulfil any regulatory function, and therefore, any changes in the gene expression profile detected by comparative transcriptomic analysis will affect processes related only to the formation of aggregates of these proteins.

Notably, our proposed experimental model system for the study of human NDs, based on the analysis of aggregate formation of virus capsid proteins in yeast cells, has not been investigated in specific experiments, apart from our pilot experiments with insect densovirus capsid protein, discussed in this review. Moreover, it is known that capsid proteins of various virus types, when expressed in yeast cells, can form not amorphous aggregates and/or amyloid structures but virus-like particles—prototypical capsids consisting of only one type of protein. See, e.g., [212]. Moreover, viral factories (viral inclusions) are known to be structures in which viral particle formation, rather than utilization of viral capsid proteins, occurs.

At the same time, we aimed to show in this review that there are similarities between the viral factories and the inclusion bodies formed in human cells in NDs, as described in many studies. The key to the formation of both structures is protein aggregation, which leads to different outcomes but underlies both processes. The molecular mechanisms that induce the aggregation of specific proteins are still unknown. We hope that the line of research we are proposing will make it possible to identify the regulatory mechanisms that lead to the induction of aggregation of a specific type of protein, thus ensuring progress in the study of NDs.

## Figures and Tables

**Table 1:** Characteristic aggregating proteins and associated genes of neurodegenerative diseases.

Neurodegenerative Disease	Aggregating Proteins	Associated Genes
Alzheimer’s disease	Tau, Aβ	*APP* (Aβ), *PSEN1*, *PSEN2*
Tauopathies	Tau	*MAPT* (*tau*)
Parkinson’s disease and synucleinopathies	α-synuclein	*SNCA* (*α-syn*), *PINK1*, *PARK7/DJ1*, *PRKN/Parkin*, *ATP13A2*, *VPS35*, *LRRK2*, *GBA*
Huntington’s disease	Huntington (Htt), PolyQ	*Htt*
Spinocerebellar ataxias	Ataxin, PolyQ	*Sca 1–3*, *Sca 7*
Amyotrophic lateral sclerosis	TDP43, tau, SOD1, FUS, DPRs	*SOD1*, *FUS*, *TDP43*, *C9ORF72*, *UBQLN2*, *VCP*, *TBK1*, *ANXA11*, *PFN1*, *KIF5A*, *VAPB*, *HNRNPA1*, *SQSTM1*, *NEK1*, *OPTN*, *TUBA4A*
Frontotemporal dementia		*GRN*, *MAPT*, *C9ORF72*, *VCP*, *CHMP2B*, *SQSTM1*, *UBQLN2*, *TBK1*
Traumatic brain injury	Tau, Aβ	?
Prion disease	PrP	*PRNP*

**Table 2:** Model substrates used to monitor inclusion formation and localization of misfolded proteins in *S. cerevisiae*.

Misfolding Protein	Origin	Experimental Conditions
Luciferase	*Photinus pyralis*	Heat shock
Guk 1-7 (guanylate kinase temperature-sensitive) temperature-sensitive	*Saccharomyces cerevisiae*	Heat shock
Gus 1-3 (glutamyl-tRNA synthetase) temperature-sensitive	*Saccharomyces cerevisiae*	Heat shock
Pro 3-1 (delta 1-pyrroline-5-carboxylate reductase) temperature-sensitive	*Saccharomyces cerevisiae*	Heat shock
Ubc9ts (SUMO-conjugating E2 enzyme) temperature-sensitive	*Saccharomyces cerevisiae*	Heat shock
VHL (von Hippel–Lindau tumor suppressor) temperature-sensitive	*Homo sapiens*	Constitutively unfolded in yeast cells (absent binding partner)
ΔssCPY* (mutated form of carboxypeptidase Y)	*Saccharomyces cerevisiae*	Constitutively unfolded
tGnd1 (truncated phosphogluconate dehydrogenase)	*Saccharomyces cerevisiae*	Constitutively unfolded
DegAB (the entire Ndc10 degron)	*Saccharomyces cerevisiae*	Constitutively unfolded
